# Hospital Expenditure and Its Reduction

**Published:** 1905-01-21

**Authors:** Sydney Holland


### Editor's Letter-Box.

[Our Correspondents are reminded that prolixity is a great bar to publication, and that brevity of style and conciseness of statement greatly facilitate early insertion.]

### HOSPITAL EXPENDITURE AND ITS REDUCTION.

Mr. Sydney Holland writes: Would Sir Henry Burdett kindly give us the figures upon which he forms the estimate that £60,000 a year would give £300 a year to the "majority of the staff of a great hospital." This is, of course, a very different estimate to that which he first made in the *Daily Mail* which was "at a rough estimate it would cost the London Hospitals £60,000 a year to pay the doctors—or say about £6 10s. per occupied bed." Not a word here about "majority of the staff" or "at a great hospital." But until we know the data upon which the estimate is made, it is difficult to discuss it or to attach much weight to it.

[Great questions cannot be discussed to the public advantage on debating society lines. It is due to a great subject that anybody who enters into a discussion upon it should carefully master the principles involved and deal with them seriously. Mr. Holland is clearly wrong in speaking of "a very different estimate" (see p. 305). It will be seen Sir Henry merely illustrated his meaning by fixing a maximum of £300. Mr. Holland, as the extracts from the correspondence in the *Daily Mail* showed, avoided the principles and strove to narrow the issue to a premature discussion of details—premature because, until the principle of the abolition of free medical service at the voluntary hospitals is determined, the details as to what sum shall be paid to the staff at each hospital, and to each member of that staff, must necessarily be left in abeyance. This is the view which is clearly indicated by Sir Henry Burdett in his method of dealing with the subject, and is manifestly correct. We gladly offer the hospitality of our columns to Mr. Sydney Holland to discuss the principles involved, but for the reasons stated, we feel no useful purpose can be fulfilled by a correspondence limited to narrow issues and matters of detail. Mr. Holland, indeed, seems to be fully aware that "readers no doubt tire of this" latter method.—Ed. The Hospital.]

